# Systematic review and meta-analysis of the proportion of *Campylobacter* cases that develop chronic sequelae

**DOI:** 10.1186/1471-2458-14-1203

**Published:** 2014-11-22

**Authors:** Jessica Keithlin, Jan Sargeant, M Kate Thomas, Aamir Fazil

**Affiliations:** Centre for Public Health and Zoonoses, University of Guelph, Guelph, Ontario Canada; Department of Population Medicine, Ontario Veterinary College, Guelph, Ontario Canada; Centre for Food-borne, Environmental and Zoonotic Infectious Diseases, Public Health Agency of Canada, Guelph, Ontario Canada; Laboratory for Foodborne Zoonoses, Public Health Agency of Canada, Guelph, Ontario Canada

**Keywords:** Foodborne disease, Campylobacter, Systematic literature review, Meta-analysis, Chronic sequelae, Reactive arthritis, Irritable bowel syndrome, Inflammatory bowel disease, Guillain Barré syndrome, Haemolytic uraemic syndrome

## Abstract

**Background:**

Understanding of chronic sequelae development after *Campylobacter* infection is limited. The objective of the study was to determine via systematic review and meta-analysis the proportion of *Campylobacter* cases that develop chronic sequelae.

**Methods:**

A systematic review of English language articles published prior to July 2011 located using Pubmed, Agricola, CabDirect, and Food Safety and Technology Abstracts. Observational studies reporting the number of *Campylobacter* cases that developed reactive arthritis (ReA), Reiter’s syndrome (RS), haemolytic uraemic syndrome (HUS), irritable bowel syndrome (IBS), inflammatory bowel disease (IBD) ,Guillain Barré syndrome (GBS) or Miller Fisher syndrome (MFS) were included. Data extraction through independent extraction of articles by four reviewers (two per article). Random effects meta-analysis was performed and heterogeneity was assessed using the I^2^ value. Meta-regression was used to explore the influence of study level variables on heterogeneity.

**Results:**

A total of 31 studies were identified; 20 reported on ReA, 2 reported on RS, 9 reported on IBS, 3 studies reported on IBD, 8 reported on GBS, 1 reported on MFS and 3 reported on HUS. The proportion of *Campylobacter* cases that developed ReA was 2.86% (95% CI 1.40% - 5.61%, I^2^ = 97.7%), irritable bowel syndrome was 4.01% (95% CI 1.41% - 10.88%, I^2^ = 99.2%). Guillain Barré syndrome was 0.07% (95% CI 0.03% - 0.15%, I^2^ = 72.7%).

**Conclusions:**

A significant number of *Campylobacter* cases develop a chronic sequela. However, results should be interpreted with caution due to the high heterogeneity.

**Electronic supplementary material:**

The online version of this article (doi:10.1186/1471-2458-14-1203) contains supplementary material, which is available to authorized users.

## Background

Globally *Campylobacter* causes acute gastrointestinal illness (AGI) in millions of people each year. In the United States there are a reported 43,698 cases of laboratory confirmed *Campylobacter spp.* annually with estimates of 845,024 (90% CI 337,031 – 1,611,083) cases yearly after adjusting for under-reporting and under-diagnosis [1]. In Canada, annual incidence rates of laboratory-confirmed illness are estimated at 28.4 cases of campylobacteriosis per 100 000 [2], with actual case counts estimated to be 20–50 times greater than what is laboratory confirmed [3]. Acute complications include bacteraemia, hepatitis, and pancreatitis. Potential long term health effects, or chronic sequelae, such as Guillain-Barré syndrome (GBS), reactive arthritis (ReA) or Reiter’s syndrome (RS), post-infectious irritable bowel syndrome (IBS) and inflammatory bowel disease (IBD) have also been associated with infection [4, 5].

The most common species of *Campylobacter* associated with human illness are *Campylobacter jejuni* and *Campylobacter coli*[5]. *Campylobacter* bacteria are naturally present in digestive tracts of animals such as swine and poultry [6, 7], which present the opportunity for the bacteria to enter the food system. In addition to foodborne infections*, Campylobacter* transmission has been linked to travel, contaminated water, direct animal contact and person to person transmission [8].

Currently, burden of disease estimates that incorporate the proportion of cases of *Campylobacter* that develop severe, long term complications are based on limited information. For example, in Canada the Ontario Burden of Infectious Disease Study (ONBOIDS) [9] attempted to capture the effects of these complications in estimates for disease burden. However, these estimates were based on a single study from the Netherlands [10]. From the United States, updated estimates on the burden associated with foodborne illness did no capture chronic sequelae and considered only hospitalization and deaths [1]. Because of the potential severity and chronic nature of post infection sequela, to develop true estimates for the burden of disease for *Campylobacter*, an accurate estimate for the number of cases that develop sequela is needed.

Systematic reviews are an established approach to identifying and summarizing a body of literature associated with a topic area [11]. Meta-analysis (the formal statistical pooling of data from multiple studies), can be used to develop summary estimates for proportions. Meta-regression allows for the exploration of study level factors that influence outcomes to assist with the interpretation of these estimates [12]. The usefulness of findings from recent systematic reviews for foodborne disease or chronic sequela are limited as they combine multiple pathogens [13], review the literature but are not formal systematic reviews [14–16] or do not present an estimate for the proportion of cases of *Campylobacter* that will develop a sequelae [17–19]. Many of the reviews report potential reasons for differences in estimates for chronic sequelae development between studies [13, 17, 18] however exploration of the impact of these factors on the results reported in studies is limited.

The purpose of the systematic review and meta-analysis presented here was to develop an estimate for the proportion of cases of *Campylobacter* that develop ReA or RS, GBS or Miller Fisher syndrome (MFS), IBS, IBD including ulcerative colitis (UC) and Crohn’s disease (Crohn’s), and haemolytic uraemic syndrome (HUS) and use meta-regression to explore study level factors that might contribute to the range of outcomes reported. Due to the high incidence of infection and potential severity of these long term complications, systematically determining these proportions from multiple published estimates would assist in understanding the true burden of disease associated with *Campylobacter.*

## Methods

### Literature search and inclusion–exclusion criteria/data variables

The following search terms were entered into four electronic databases (Pubmed, Agricola, CabDirect, and Food Safety and Technology Abstracts) to identify studies related to chronic sequelae of *E. coli* O157, *Salmonella* and *Campylobacter*; (‘*Escherichia coli* O157′, or, ‘O157′, ‘VTEC’, ‘STEC’, ‘O157:H7’ or *Salmonella* or *Campylobacter)* and (‘sequel*’, ‘long-term’, ‘long term’, ‘chronic ‘, ‘Guillain*’, ‘HUS’, ‘hemolytic uremic syndrome’, ‘haemolytic uraemic syndrome’, ‘hemorrhagic uremic syndrome’, ‘haemorrhagic uraemic syndrome’, ‘Reiter*’, ’complication*’, ‘arthritis’, ‘irritable bowel syndrome’, ‘IBS’, ‘post infectious irritable bowel syndrome’ or ‘inflammatory bowel disease’), without language restrictions to identify citations from prior to July 2011. The systematic review met the criteria outlined in the Preferred Reporting Items for Systematic Reviews and Meta-Analyses (PRISMA) 2009 guidelines. (See Additional file 1).

Three levels of screening were performed. The first and second rounds of screening were based on titles and abstracts only while the third round consisted of a review of full text articles. The first screening was performed by a single reviewer, and excluded references that did not contain information on the pathogens of interest or those that were not the study designs of interest (included observational studies only). The second screening was performed independently by two reviewers per reference with differences solved by consensus. The purpose was to restrict the results to more specific pathogen subtypes (non-typhoidal *Salmonella* and *Campylobacter* species except for *Campylobacter pylori)* and chronic sequelae (ReA, RS, IBD, IBS, GBS, MFS, and GBS). Studies on *Campylobacter pylori* were excluded as it has been more recently reclassified as *Helicobacter pylori*. The third level of screening identified those publications that contained the information necessary to answer the research question and data extraction was performed on those that met the criteria. Screening and data extraction were performed by four different researchers with two researchers independently reviewing each full text article. Conflicts were resolved via discussion to achieve consensus, with any remaining disagreements resolved by a third reviewer. Included studies were observational studies that provided details on the number of cases of *Campylobacter* that developed one or more of the chronic sequelae of interest. Studies reporting the opposite relationship, i.e. the number of cases of sequelae with evidence of past *Campylobacter* exposure, were excluded.

Data were extracted on population (years and season for data collection, country and age range and gender distribution of *Campylobacter* cases), *Campylobacter* species, study directionality (retrospective vs. prospective), source of data (surveillance vs. outbreak vs. hospitalized cases), sequelae status prior to illness with *Campylobacter*, categories describing both the *Campylobacter* diagnosis and the sequelae diagnosis, the length of time between *Campylobacter* infection and sequelae diagnosis (follow-up time) and outcomes (number of cases of *Campylobacter*, number of *Campylobacter* cases who developed chronic sequelae). Prospective studies were those where cases of *Campylobacter* were identified and the assessment for sequelae occurred at a time point in the future. Retrospective studies were those where both the identification as a case of *Campylobacter* and sequelae diagnosis had already occurred prior to the study initiation. Diagnosis of *Campylobacter* was categorized as confirmed or probable based on the description of diagnostic methods provided in each publication. Confirmed cases of *Campylobacter* were those where cases were identified by culture, serology or DNA-based tests and probable cases were those identified as a case based on the clinical case definition given in the study. Diagnoses of the sequelae were categorized as assessment by specialist, physician diagnosed/taken from medical records or self-reported. Season was classified as fall (September to November), winter (December to February), spring (March to May) and summer (June to August) in the northern hemisphere. In the southern hemisphere they were classified as fall (March through May), winter (June through August), spring (September through November) and summer (December to February). For the analysis, years of data collection were classified as decade based on when data collection began.

After data extraction, studies on outcomes specific to *Campylobacter* were identified for inclusion in this review. Each combination of *Campylobacter* or sequelae diagnosis was considered as a separate outcome measure as some studies reported multiple methods of diagnosing both *Campylobacter* ( e.g. a study reporting both probable and confirmed cases) and the sequelae (e.g. a study reporting self-reported and specialist confirmed cases of the sequelae) as well as multiple data sources (e.g. both outbreak associated and hospitalized cases). Based on these various classifications, it was possible to calculate multiple estimates from the same study for the proportion of cases of *Campylobacter* that developed a sequela. Therefore the term “outcome measure” was used to describe the probability of a case of *Campylobacter* developing a chronic sequela for a specific classification.

### Statistical analysis

The primary outcome was the proportion of people with *Campylobacter* who developed a specific chronic sequela. This was calculated as the number of persons developing a sequela divided by the total number of cases of *Campylobacter*. Standard errors and confidence intervals for a single proportion were derived. Prior to analysis adjusted proportions were calculated using a logit transformation [20].


Where p is the proportion of people developing the sequela and n is the total number of cases of *Campylobacter*.

All statistical analyses were performed in Stata Version 12 (Statacorp, 2012). Meta-analysis was performed using a random effects model and the DerSimonian and Laird method [21]. Heterogeneity was assessed using the I^2^ value [22]. A count of 0.5 was added to or subtracted from the number of sequela cases to those reporting a chronic sequelae outcome of 0% or 100% respectively, to allow for inclusion in the meta-analysis. Meta-regression was used to explore potential sources of heterogeneity if the I^2^ value was higher than 25% and if greater than 10 outcome measures were present for the sequela of interest [12]. The source of data, the method of diagnosing *Campylobacter*, *Campylobacter* species, method of diagnosing the sequelae, country, study directionality, group size (total number of cases included in study), decade of data collection and follow-up time were considered as explanatory variables.

Categorical variables representing group size and follow up time were generated for inclusion in the meta-regression. Group size was divided into extra small (n < 100), small (n = 100 to 500), medium (n = 501 to 1000), large (n = 1001 to 9999) and extra-large (n ≥ 10000). Follow-up time was divided into four categories; less than a month (30 days), over 30 days to less than 3 months (90 days), more than 90 days to less than a year, and a year (365 days) or longer. Study date was classified by the earliest decade for data collection. Factors were only included in the meta-regression if there was variation in the factor among studies. Univariable analysis was performed to test for significance. Variables with a p-value of ≤ 0.05 in the univariable analyses were included in a backwards multivariable model and those that remained significant (p ≤ 0.05) were further explored with subgroup meta-analysis. Meta-regression was performed using logit transformed outcomes and logit transformed within-study standard errors.

### Assessment of reporting of factors related to internal and external validity

Information on reporting of factors related to internal validity (risk of bias) and external validity (generalizability) were extracted to allow for further exploration. Ten criteria were extracted. Factors related to internal validity were study directionality, the source of data, method of diagnosis for both the pathogen and sequelae, follow-up time, and reporting the specific criteria used for the sequelae diagnosis. The definitions for sequelae diagnosis were divided into two categories; the method of diagnosis (physician vs. self-reported vs. other) and whether specific diagnostic criteria were. Factors related to external validity were the reporting of relevant population information (country, gender distribution and age range of *Campylobacter* cases).

## Results

### Systematic review

#### Study selection

**Figure 1:**
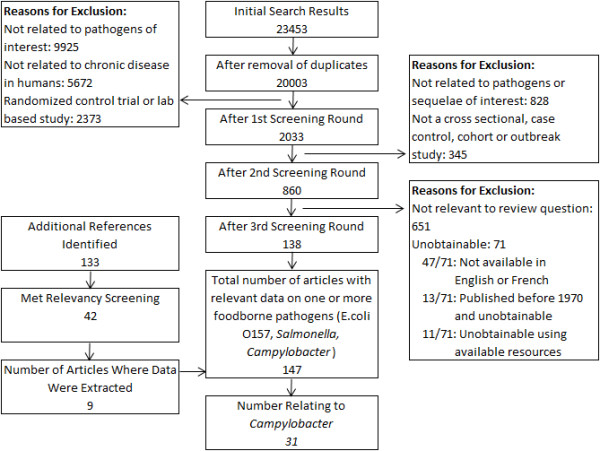
**Results from the literature search for studies relating to chronic sequelae associated with** ***Campylobacter*** **published prior to July 2011.**

Of the 20048 unique citations identified, 860 required review of the full text article of which 651 were deemed inapplicable to the research question, 47 studies were not available in English or French, 24 were inaccessible (Figure 1). After screening, 147 studies underwent data extraction of which 31 contained relevant information on *Campylobacter* and chronic sequelae.

### Reactive arthritis

#### Study descriptions

Of the 31 studies investigating *Campylobacter* and chronic sequelae (Table 1), 20 provided data on ReA. The 20 studies were from nine countries with almost all (8/9) from Europe. Sixty percent (12/20) were based on surveillance data and of those the majority (58%, 7/12) were prospective in design. Of the five outbreak studies, 60% (3/5) were waterborne.

**Table 1:** **Population characteristics for studies relating to selected chronic sequelae of** ***Campylobacter*** **published before July 2011**

First author, year, reference number	Country	Sequelae~	Age range (for ***Campylobacter*** cases)	% female (for ***Campylobacter*** cases)	Source of data	Study directionality	Outbreak source	Date for data collection	Season
Pitkanen, 1981 [23]	Finland	ReA	All ages	46	Hospitalized cases	Other^	NA	1978-1980	All
Wang, 2008 [24]	Taiwan	GBS	Youth+	30	Hospitalized cases	Other	NA	2000-2006	All
Short, 1982 [25]	UK	ReA	NR	NR	Hospitalized cases	Other	NA	1979	Summer/Fall
Rowe, 1991 [26]	Canada	HUS	Youth	60	Hospitalized cases	Prospective	NA	1985 - 1988	All
Petersen, 1996 [27]	Denmark	ReA	All ages	49	Hospitalized cases	Prospective	NA	1991-1993	All
Saps, 2008 [28]	USA & Italy	IBS	Youth	48	Hospitalized cases	Prospective	NA	2006	Summer/Fall
Pitkanen, 1983 [29]	Finland	ReA	All ages	47	Hospitalized cases	Retrospective	NA	1978-1981	All
Spiller, 2000 [30]	UK	IBS	Adults	60	NR	Prospective	NA	NR	NR
Spence, 2007 [31]	New Zealand	IBS	NR	NR	Other	Prospective	NA	Pre-2006	All
Melby, 1990 [32]	Norway	ReA	All ages	48.5	Outbreak in community	Prospective	Waterborne	Pre-1990	Spring/Summer
Bremmel, 1991 [33]	Sweden	ReA	NR	55	Outbreak in community	Prospective	NR	1981	Fall
Gardner, 2011 [34]	USA	GBS	All ages	1	Outbreak in community	Prospective	Food - vegetable	2008	Summer/Fall
Eastmond, 1983 [35]	Scotland	ReA	NR	NR	Outbreak in community	Retrospective	Food - dairy	1979	Winter
McCarthy, 1999 [36]	Sweden	GBS	NR	NR	Outbreak in community	Retrospective	Waterborne	1980,1994, 1995	Fall, Spring, Spring
Locht, 2002 [37]	Denmark	ReA	Adults	56	Surveillance*	Other	NA	1997-2000	All
Helms, 2006 [38]	Denmark	GBS, IBD, IBS, HUS, ReA	All ages	50	Surveillance	Retrospective	NA	1991 - 1999	All
Kosunen, 1981 [39]	Finland	ReA, RS	NR	NR	Surveillance	NR	NA	1978 - 1979	All
Ponka, 1984 [40]	Finland	ReA	All ages	NR	Surveillance	Prospective	NA	1978 - 1991	All
Schiellerup, 2008 [41]	Denmark	ReA	Adults	57.1	Surveillance	Prospective	NA	2002-2003	All
Dunlop, 2003 [42]	England	IBS	Adults	nr	Surveillance	Prospective	NA	1999-2002	All
Hannu, 2002 [43]	Finland	ReA	All ages	59	Surveillance	Prospective	NA	1997-1998	All
Doorduyn, 2008 [44]	Netherlands	ReA, RS, GBS, MFS, IBS	NR	nr	Surveillance	Prospective	NA	2002-2003, 2005	NR
Moss-Morris 2006, [45]	New Zealand	IBS	Adults	56	Surveillance	Prospective	NA	2002-2003	All
Thornley, 2001 [46]	UK	IBS	Adults	NR	Surveillance	Prospective	NA	1997	Spring/Summer
Townes, 2008 [47]	USA	ReA	All ages	50	Surveillance	Prospective	NA	2002-2004	All
Jess, 2011 [48]	Denmark	Crohn’s, UC	All ages	50	Surveillance	Retrospective	NA	1992 - 2008	All
Gumpel, 1981 [49]	England	ReA	All ages	nr	Surveillance	Retrospective	NA	1978	All
Tam, 2006 [50]	UK	GBS	NR	NR	Surveillance	Retrospective	NA	1991 - 2001	All
Scoenberg-Norio, 2010 [51]	Finland	ReA	All ages	48.3	Surveillance	Prospective	NA	2002	Summer/Fall
Ternhag, 2008 [52]	Sweden	GBS, ReA, HUS, IBS, UC, Crohn’s	All ages	51	Surveillance	Retrospective	NA	1997-2004	All
McCarthy, 2001 [53]	Sweden	GBS	All ages	NR	Surveillance	Retrospective	NA	1987-1995	All

^Other included combinations of both approaches.

*Population surveillance includes laboratory and notifiable disease registries, sporadic cases and other population surveillance. NR = Not Reported.

+Youth were those younger than 18. Adults were ≥ 18 years.

~ReA = reactive arthritis; RS = Reiter’s syndrome; GBS = Guillain Barré syndrome; MFS = Miller-Fisher syndrome; HUS = haemolytic uraemic syndrome; IBS = irritable bowel syndrome; IBD = inflammatory bowel disease; UC = ulcerative colitis; Crohn’s = Crohn’s disease.

#### Outcome measure

**Figure 2:**
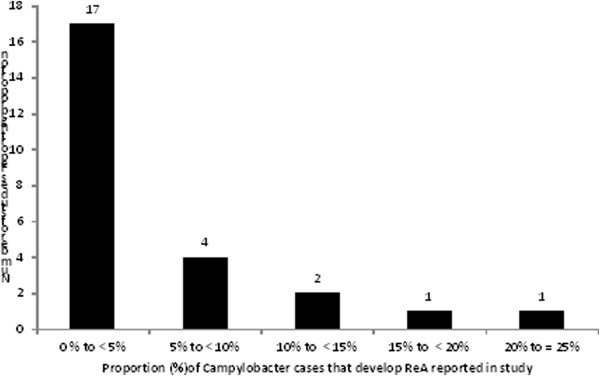
**Distribution of estimates of the proportion of** ***Campylobacter*** **cases that develop reactive arthritis from studies published prior to July 2011.**

There were 25 outcome measures described, with two studies [35, 43] describing multiple outcome measures (Table 2). The proportions reported most often for the development of ReA after *Campylobacter* infection were less than 5% (Figure 2). Surveillance based studies ranged from 13 to 57425 confirmed cases of *Campylobacter*, with the probability of a case of *Campylobacter* developing ReA ranging from 0% to 24%. Outbreak studies ranged from 42 to 350 cases with the probability of developing ReA ranging from 0% to 1.5% for confirmed cases and 0.6% to 2.5% for probable cases. For hospital based studies case numbers ranged from 41 to 188 cases with the probability of developing ReA ranging from 1.2% to 5.36%.

**Table 2:** **Outcome variables organized by chronic sequelae for studies relating to** ***Campylobacter*** **published prior to July 2011**

First author, year, reference number	Species	Sequelae negative prior to diagnosis with ***Campylobacter*** ?	Time from ***Campylobacter*** diagnosis to evaluation for chronic sequelae (Days)	Diagnosis of ***Campylobacter***	Diagnosis of sequelae	Number of people with ***Campylobacter***	Number developing sequelae	Outcome
**ReA**								
Ternhag, [52]	spp.	NR	365	NR	Physician/medical records^	57425	15	0.03%
Schoenberg-Norio, [51]	*jejuni*	NR	60	Confirmed+	Physician/medical records	201	8	3.98%
Townes, [47]	NR	All were disease negative	42	Confirmed	Combination	2384	33	1.38%
Kosunen, [39]	*jejuni*	All were disease negative	365	Confirmed	Physician/medical records	342	8	2.34%
Petersen, [27]	mix	NR	NR	Confirmed	Physician/medical records	41	0	0%
Short, [25]	*jejuni*	NR	240	Confirmed	Physician/medical records	15	0	0%
Hannu, [43]	mix	All were disease negative	60	Confirmed	Physician/medical records	609	45	7.39%
Hannu, [43]	mix	All were disease negative	60	Confirmed	Combination	609	52	8.54%
Hannu, [43]	*jejuni*	All were disease negative	60	Confirmed	Specialist	535	37	6.92%
Hannu, [43]	*coli*	All were disease negative	60	Confirmed	Specialist	61	8	13.11%
Hannu, [43]	Undetermined	All were disease negative	60	Confirmed	Specialist	13	0	0%
Melby, [32]	*jejuni*	NR	42	Probable	Self-reported disease status	159	1	0.63%
Pitkanen, [23]	*jejuni*	NR	NR	Confirmed	Self-reported disease status	56	3	5.36%
Locht, [37]	mix	All were disease negative	28	Confirmed	Self-reported disease status	173	27	15.61%
Schiellerup, [41]	NR	No – excluded~	60	Confirmed	Self-reported based on validated scale	1003	131	13.06%
Hannu, [54]	*jejuni*	All were disease negative	90	Probable	Specialist	350	9	2.57%
Pitkänen, [29]	*jejuni*	All were disease negative	NR	Confirmed	Combination	188	9	4.79%
Helms, [38]	NR	All were disease negative	365	Confirmed	Physician/medical records	17991	22	0.12%
Ponka, [40]	*jejuni*	NR	NR	Confirmed	Self-reported disease status	283	6	2.12%
Doorduyn, [44]	NR	NR	1080	Confirmed	Self-reported disease status	434	20	4.61%
Eastmond, [35]	*jejuni*	All were disease negative	90	Confirmed	Physician/medical records	88	1	1.14%
Eastmond [35]	*jejuni*	All were disease negative	90	Confirmed	Physician/medical records	42	0	0%
Melby, [55]	mix	NR	NR	Probable	Self-reported disease status	77	2	2.60%
Gumpel, [49]	NR	NR	NR	Confirmed	Physician/medical records	33	8	24.24%
Bremell, [33]	NR	NR	24	Confirmed	Self-reported disease status	66	1	1.52%
**Reiter’s**								
Kosunen, [39]	*jejuni*	All were disease negative	Up to 365	Confirmed	NR	342	1	0.29%
Doorduyn, [44]	NR	NR	1080	Confirmed	Self-reported disease status	457	0	0%
**IBS**								
Ternhag, [52]	spp	NR	365	NR	Physician/medical records	57425	15	0.03%
Spence, [31]	spp	All were disease negative	180	Confirmed	Self-reported based on validated scale	620	49	7.90%
Thornley, [46]	spp.	All were disease negative	180	Confirmed	Self-reported based on validated scale	188	17	9.04%
Spiller, [30]	NR	NR	365	Confirmed	Self-reported based on validated scale	31	4	12.90%
Helms, [38]	NR	All were disease negative	365	Confirmed	Physician/medical records	17991	161	0.89%
Doorduyn. [44]	NR	No – included	1080	Confirmed	Self-reported disease status	457	12	2.63%
Saps, [28]	NR	NR	180	Confirmed	Self-reported	6	1	16.67%
Dunlop, [42]	*jejuni*/*coli*	All were disease negative	90	Confirmed	Self-reported based on validated scale	747	103	13.79%
Moss-Morris, [45]	NR	All were disease negative	90	Confirmed	Self-reported based on validated scale	775	83	10.71%
Moss-Morris, [45]	NR	All were disease negative	180	Confirmed	Self-reported based on validated scale	748	59	7.89%
**IBD**								
Helms, [38]	NR	All were disease negative	365	Culture	Physician/Medical records	17991	72	0.40%
**UC**								
Ternhag, [52]	spp.	NR	365	NR	Physician/Medical records	57425	42	0.07%
Jess, [48]	NR	All were disease negative	Up to 16 years	Confirmed	Physician/Medical records	49420	223	0.45%
**Crohn’s**								
Ternhag, [52]	spp.	NR	365	NR	Physician/Medical records	57425	27, 83	0.05%, 0.17%
Jess, [48]		All were disease negative	Up to 16 years	Confirmed	Physician/Medical records	49420	83	0.17%
**GBS**								
Ternhag, [52]	spp.	NR	90	NR	Physician/medical records	57425	13	0.02%
Gardner, [34]	*jejuni*	All were disease negative	NR	Probable	Physician/medical records	98	1	1.02%
McCarthy, [36]	*jejuni*	No – excluded	180	Probable	Physician/medical records	8086	0	0%
Wang, [24]	*coli*	NR	NR	Confirmed	Physician/medical records	24	0	0%
Wang, [24]	*jejuni*	NR	NR	Confirmed	Physician/medical records	80	0	0%
Wang [24]	mix	NR	NR	Confirmed	Physician/medical records	104	0	0%
McCarthy, [53]	NR	All were disease negative	180	Confirmed	Physician/medical records	29563	9	0.03%
Helms, [38]	NR	All were disease negative	365	Confirmed	Physician/medical records	17991	6	0.03%
Tam, [50]	NR	All were disease negative	60	NR	Physician/medical records	15587	3	0.02%
Doorduyn, [44]	NR	NR	1080	Confirmed	Physician/medical records	457	0	0%
**MF**								
Doorduyn, [44]	NR	NR	1080	Confirmed	Self-reported disease status	457	0	0%
**HUS**								
Ternhag, [52]	spp.	NR	90	NR	Physician/Medical records	57425	2	0%
Helms, [38]	NR	All were disease negative	90	Confirmed	Physician/Medical records	17991	1	0.01%
Rowe, [26]	NR	All were disease negative	NR	Confirmed	Physician/Medical records	72	0	0%

NR = Not Reported.

^Medical Records/Physician includes those hospitalized for sequelae or diagnosed by a physician.

+ Confirmed for *Campylobacter* are those confirmed by culture, DNA based tests or serology. Probable cases were based on case definition given in study.

~No – excluded; *Campylobacter* cases with previous medical history of related sequelae were excluded from analysis. No-included; *Campylobacter* cases with previous medical history of related sequelae were not excluded from analysis.

### Assessment of internal and external validity

Thirty percent of studies (6/20) did not report the time from *Campylobacter* diagnosis to diagnosis of the sequelae (Table 2). Fifty-five percent (11/20) of studies did not report whether or not ReA cases were disease negative (did not have arthritis) at the time of diagnosis of campylobacteriosis. Age range and gender distribution were missing from 35% (7/20) and 40% (8/20) of studies, respectively. Data source was reported in all studies however in one study it was not possible to determine study directionality. The method for *Campylobacter* diagnosis was reported in all studies but one, and the methods of sequelae diagnosis (physician vs. self-reported) were reported in all studies. However, the specific diagnostic criteria for sequelae diagnosis were not provided for 40% (8/20) of studies.

### Meta-analysis/meta-regression

**Figure 3:**
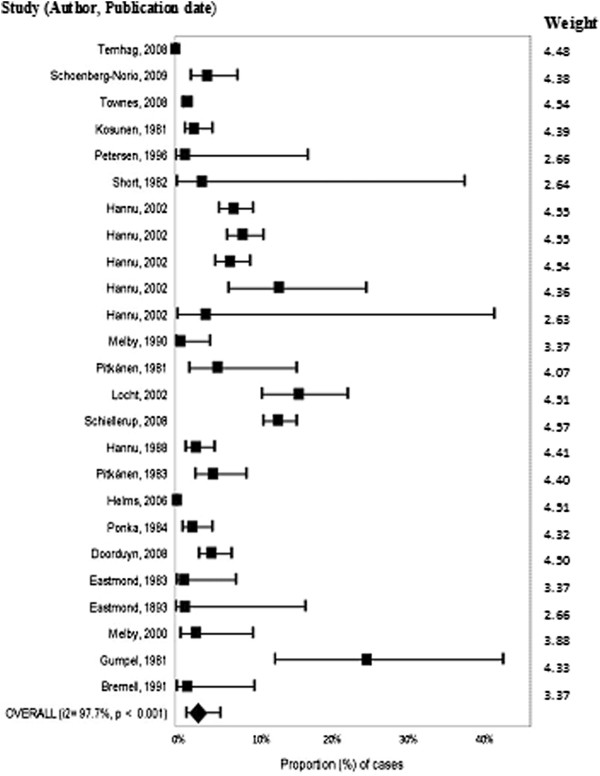
**Forest plot of the proportion of cases of reactive arthritis from studies published before July 2011.**

A total of 25 outcome measures were included. The overall summary estimate of the proportion of cases of *Campylobacter* that developed ReA was 2.86% (95% CI 1.4% - 5.61%). Heterogeneity was high at 97.7% (Figure 3). Due to high heterogeneity, exploration of factors influencing the outcome measure was performed using meta-regression.

Variables found to have a significant contribution to the heterogeneity in univariable meta-regression were group size (p = 0.015), follow-up time (p = 0.005) and *Campylobacter* species (p = 0.014). Due to the limited number of studies providing information for all of these factors (n = 12) and evidence of non-independence within studies (all of the medium size outcome measures were from the same study), multivariable analysis incorporating all three categories was not possible. Therefore all three of these factors were explored individually with subgroup meta-analysis.

Based on follow-up time, the proportion of cases of *Campylobacter* that developed ReA ranged from 0.44% (95% CI 0.04% - 4.77%, I^2^ = 98.9%) in studies following patients for a year or more to 5.92% (95% CI 3.51% - 9.81%, I^2^ = 94.2%) in studies with a follow up of less than 90 days (Table 3). A lower proportion of cases of *Campylobacter jejuni* developed ReA (3.29%; 95% CI 2.18% - 4.93%, I^2^ = 58.8%) versus those that were a mix of or unidentified species (8.27%; 95% CI 5.41% - 12.46%, I^2^ = 70.4%) (Table 3). Group size demonstrated a large range, in groups with case numbers of 10 000 or more the summary estimates for ReA was 0.06% (95% CI 0.01% - 0.26%, I^2^ = 95.3%) of *Campylobacter* cases developing ReA versus 4.55% (95% CI 1.99% - 10.07%, I^2^ = 66.7%) in those studies with under 100 cases (Table 3).

**Table 3:** **Results of subgroup meta- analyses for** ***Campylobacter*** **and reactive arthritis from studies published prior to July 2011**

Variable	Summary estimate	Lower 95% CI	Upper 95% CI	I ^2^	Number of outcome measures
***Overall Estimate***	2.86%	1.4%	5.61%	97.7%	25
***Follow Up Time***					
Over a year	0.44%	0.04%	4.77%	98.9%	4
> 3 months to < year	3.42%	14.84%	12.92%	91.0%	4
< 3 months	5.92%	3.51%	9.81%	94.2%	11
***Species***					
*Jejuni*	3.29%	2.18%	4.93%	58.8%	11
Mix/undetermined*	8.27%	5.41%	12.46%	70.4%	6
***Group Size***					
Extra small	4.55%	1.99%	10.07%	66.7%	10
Small	3.73%	1.99%	6.33%	87.1%	8
Medium	^	^	^	^	3
Large	4.41%	0.45%	32.02%	99.3%	2
Extra large	0.06%	0.01%	0.26%	95.3%	2

*Included combinations of *jejuni*, *coli*, *spp* and undetermined species.

^Not calculated, no variation between variables of interest as all outcome measures were from the same study.

### Reiter’s syndrome

A closely related subtype of ReA is Reiter’s syndrome (RS) which is characterized by arthritis in combination with conjunctivitis, and urethritis [17]. Two studies reporting on ReA also reported outcome measures specific to the related sequelae of RS. The proportion of culture confirmed cases of *Campylobacter* developing RS were 0% and 0.29%.

### Irritable bowel syndrome

#### Study descriptions

Nine studies provided information on *Campylobacter* and irritable bowel syndrome (IBS) (Table 1). The nine studies were from seven different countries, with one of the studies presenting both US and Italian data. The majority (77%, 7/9), were based on surveillance data and were prospective (89%, 8/9). Follow-up times ranged from three months (90 days) to three years (1080 days).

#### Outcome measures

There were ten different outcome measures, as one study [45] included more than one outcome measure. The proportion of confirmed cases of *Campylobacter* that developed IBS ranged from 0.03% to 0.89% in those studies reporting IBS diagnosis from medical records; 7.89% to 13.79% for those diagnosed using a self-report according to a validated scale (Rome I and Rome I modified [56] criteria or Rome II [57] criteria, Gastrointestinal Symptom Rating Score [58]) and 2.63% to 16.63% in those that were self-reported cases of IBS (Table 2).

### Assessment of internal and external validity

Data on age range and gender distribution were missing from 22% (2/9) and 44% (4/9) of studies respectively. Study directionality could be determined in all studies however the source of data was missing from one (Table 2). The time between *Campylobacter* diagnosis and diagnosis of IBS was reported in all studies. However, the method of diagnosing *Campylobacter* was missing from a single study. Six studies reported on whether or not cases were disease negative for IBS prior to the study and a single study included those with a history of IBS in their outcome measure. The method of diagnosing IBS was reported in all studies but three studies did not provide the specific criteria for their diagnosis of IBS.

### Meta-analysis/meta-regression

**Figure 4:**
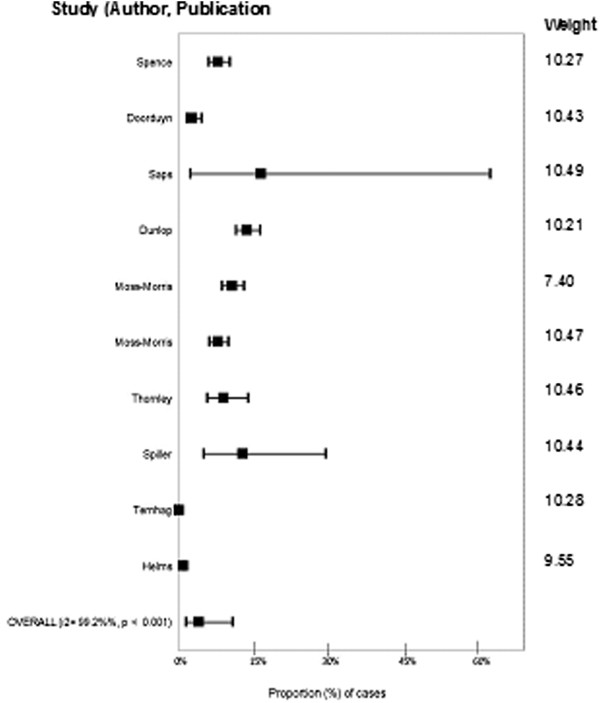
**Forest plot of the proportion of cases of** ***Campylobacter*** **that develop irritable bowel syndrome from studies published before July 2011.**

A total of 10 outcome measures were included in the analysis. The estimate for the proportion of cases of *Campylobacter* that developed IBS was 4.01% (95% CI 1.41% - 10.88%, I^2^ = 99.2%) (Figure 4). The high heterogeneity supported investigation of influencing variables with meta-regression. In univariable analyses, group size (p = 0.04), study directionality (p = 0.001) and the method of diagnosing IBS (p = 0.05) significantly contributed to heterogeneity. The limited number of studies and evidence that variables were associated (all large studies were retrospective, physician diagnosed) prevented multivariable meta-regression. Subgroup meta-analysis was therefore performed for all significant factors.

Subgroup meta-analysis demonstrated that the number of cases of *Campylobacter* that developed IBS in prospective studies was 8.64% (95% CI 6.36% - 11.66%, I^2^ = 83.7%) versus 0.15% (95% CI 0.0048% - 4.75%, I^2^ = 99.4%) in retrospective studies (Table 4). The proportion of cases of *Campylobacter* that developed IBS varied between method of sequelae diagnosis with self-reported cases (using validated scale) estimated at 9.94% (95% CI 7.90% - 12.44%, I^2^ = 73.6%) versus 5.27% (95% CI 0.84% - 26.84%, I^2^ = 68.0%) in self-reported cases and 0.15% (0.0048% - 4.75%, I^2^ = 99.4%) in cases of IBS identified from medical records. For studies with fewer than 100 cases of Campylobacter, 13.56% (95% CI 5.76% - 28.74%, I^2^ = 0%) of cases developed IBS versus 0.15% (95% CI 0.0048% - 4.75%, I^2^ = 99.4) of cases in studies with greater than 10000 participants.

**Table 4:** **Results of subgroup meta- analyses for** ***Campylobacter*** **and irritable bowel syndrome from studies published prior to July 2011**

Variable	Summary Estimate	Lower 95% CI	Upper 95% CI	I ^2^	Number of outcome measures
**Overall Estimate**	4.01%	1.41%	10.88%	99.2%	10
***Study Directionality***					
Prospective	8.64%	6.36%	11.66%	83.7%	8
Retrospective	0.15%	0.0048%	4.75%	99.4	2
***Method of Assessing Sequelae***					
Self-reported	5.27%	0.84%	26.84%	68.0%	2
Self-reported based on validated scale*	9.94%	7.90%	12.44%	73.6%	6
Based on medical records	0.15%	0.0048%	4.75%	99.4%	2
***Group Size***					
Small	13.56%	5.76%	28.74%	0.0%	2
Medium	4.96%	1.43%	15.79%	91.2%	2
Large	9.92%	7.52%	12.98%	83.6%	4
Extra large	0.15%	0.0048%	4.75%	99.4	2

*Questionnaires using self-reported responses to Rome I/Rome II criteria.

### Inflammatory bowel disease including crohn’s disease and ulcerative colitis

Inflammatory bowel disease encompasses two separate diseases, Crohn’s disease and ulcerative colitis (UC) which have closely related pathologies but are considered independent entities [57]. A single study reported the proportion of *Campylobacter* cases that developed IBD and two studies evaluated Crohn’s and UC. The proportion of culture confirmed *Campylobacter* cases developing IBD was 0.4%. For Crohn’s and UC, both studies were large retrospective surveillance studies. For confirmed cases of *Campylobacter,* 0.16% developed Crohn’s and 0.45% developed UC versus 0.047% developed Crohn’s and 0.07% developed UC in the study where the method of *Campylobacter* diagnosis was not reported.

### Guillain Barré syndrome

#### Study descriptions

Eight studies from six countries provided estimates for the number of cases of *Campylobacter* that developed GBS (Table 1). The majority (5/8) were based on surveillance data and with the data collected retrospectively (6/8). Follow up times ranged from two months to three years.

#### Outcome measures

There were ten different outcome measures (Table 2) as one study [24] reported multiple outcome measures. The proportion of confirmed cases of *Campylobacter* that developed GBS ranged from 0% to 2.08% with case numbers varying from 24 to 29563 cases of *Campylobacter* (n = 4 studies). For probable cases of *Campylobacter*, the proportion developing GBS ranged from 0.01% to 1.02% with *Campylobacter* case numbers ranging from 98 to 8086 (n = 2 studies) (Table 2).

#### Assessment of internal and external validity

Data on age range and gender distribution of *Campylobacter* cases were missing from 38% (3/8) and 50% (4/8) of studies, respectively (Table 2). The source of data and study directionality were reported in all studies. Two studies did not report the method of *Campylobacter* diagnosis. An additional two studies did not report follow-up time. Five studies reported on whether or not cases were disease negative prior to the onset of campylobacteriosis. All studies reported the method of diagnosis for the sequelae but the majority (62.5%, 5/8) did not report the specific diagnostic criteria used for their diagnosis of GBS.

#### Meta-analysis/meta-regression

**Figure 5:**
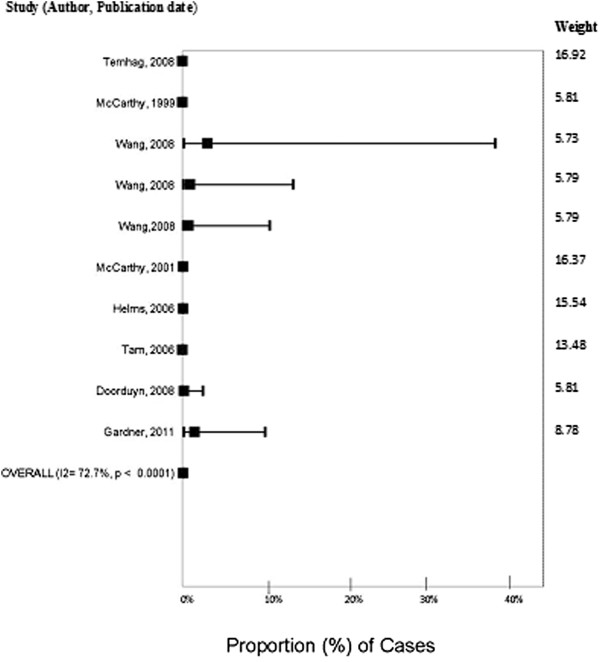
**Forest plot of the proportion of cases of** ***Campylobacter*** **that develop Guillain Barre syndrome from studies published before July 2011.**

There were ten outcome measures between the 8 studies. Five of those reported no cases of GBS. The proportion of cases of *Campylobacter* that developed GBS was estimated at 0.07% (95% CI. 0.03% - 0.15%, I^2^ = 72.7%) (Figure 5). Both group size (p = 0.001) and data source (p = 0.04) significantly contributed to heterogeneity. Multivariable meta-regression and subgroup analysis was not conducted given the limited data.

### Miller fisher syndrome

Miller-Fisher syndrome is a subtype of GBS [59]. One study reporting on GBS also reported outcome measures specific to MFS. Of the 457 cases of *Campylobacter,* none developed MFS (Table 2).

### Haemolytic uraemic syndrome

Three studies provided estimates for the proportion of cases of *Campylobacter* that developed HUS (Table 1). In a study of children hospitalized for *Campylobacter* infection, no cases of HUS were reported. The two surveillance based studies reported estimates of 0.005% and 0.003% (Table 2).

## Discussion

This study used a systematic literature search and meta-analysis to provide estimates for the number of cases of *Campylobacter* that develop the chronic sequelae of reactive arthritis, irritable bowel syndrome or Guillain Barré syndrome. Considering the high incidence of *Campylobacter* and the potential severity of its associated chronic sequelae, there was limited research available. Not enough data were available to develop summary estimates for inflammatory bowel disease and haemolytic uraemic syndrome. A strength of systematic reviews is that they attempt to systematically identify all information on a topic and present the methodology and results in a transparent and reproducible manner [11]. Even with the broad terminology used for the search terms and large number potentially relevant citations identified in this review, only 31 studies for chronic sequelae associated with *Campylobacter* infection were identified internationally. Within these 31 studies, the occurrence of chronic sequelae following infection varied greatly. For those sequela where sufficient publications were found to develop estimates using meta-analysis, the heterogeneity associated with these estimates was very high [22] indicating that the summary estimates must be interpreted with caution and within the context of those variables that were found through meta-regression to significantly influence heterogeneity.

Multiple factors were found to significantly affect heterogeneity depending on the sequela being considered. Group size was the only factor that was consistently influential between sequela estimates, with smaller studies reporting a higher proportion of sequela. This effect could be attributed in part to differences in how sequela cases are captured between smaller outbreak studies and larger surveillance or registry based studies. Large population based studies may only identify the severe cases of sequela, while individuals in smaller studies may have been followed more closely increasing the likelihood that less severe cases are captured. This could be especially relevant to sequelae such as ReA or IBS in which severity would be influenced by personal perception [60] versus Guillain Barré syndrome which is medically severe and is less likely to remain unidentified [16]. The effect of physician diagnosis versus self-report was seen in the meta-regression for IBS as studies using medical records had a much lower proportion of IBS cases than self-reported cases.

Follow-up time was significant for ReA but not IBS and GBS. For reactive arthritis individual variation in disease symptoms and recovery has been reported along with a wide range of proposed timelines for disease development [17, 61]. Because of this, understanding of disease progression (from onset to recovery) is unclear and no standard guidelines for disease duration have been accepted within the medical field; which could explain some of the variation in follow-up times used between studies. The association between follow-up time and the development of reactive arthritis suggests a peak within the first 90 days after infection. However, the association observed could be explained by differences in how studies assess a case of ReA and whether they were considering incident or prevalent cases at the time of follow-up. For example, were the cases captured after a one year follow-up prevalent cases which developed ReA within the first 90 days and did not recover or were they new incidence cases that took longer to develop and which were not present within those first 90 days. Given the minimal details provided on the specifics used to assess ReA in many of these studies, it is not possible to interpret the effect of incident vs. prevalent cases. Further investigation into disease duration is recommended as this could have significant effects on disease burden estimates. A large number of cases of ReA within the first few months after infection that recover without complication could require vastly different health care resources then long term chronic cases of ReA that require ongoing medical attention. Additionally, some sequelae cases could have developed these chronic sequelae regardless of *Campylobacter* exposure as illnesses such as IBS can develop without previous infection.

The lack of association between follow-up time and IBS and GBS diagnosis is difficult to interpret given the limited available data. For GBS half of the studies did not report follow up time, for IBS the lack of association could indicate more long term duration for the disease. However, the limited data available prevented full exploration of this theory. For all sequela, the effect of follow up time would be greatly influenced by when the follow-up questions were administered and how they were worded. For example, changing the wording from “are you currently suffering from any of the following symptoms?” to “have you ever suffered from any of the following symptoms since diagnosis with *Campylobacter*?” could yield different results. Due to the limited information reported in most publications, assessing what effect this would have on the results was not possible.

For the other factors that significantly affected heterogeneity, the limited data available also needs to be considered while interpreting the results. For directionality, which was significant for IBS, the number of studies prevents interpretation as the retrospective studies were also large surveillance studies with very low outcome estimates. Additionally, the effect of “method of diagnosis for the sequelae” should be interpreted with caution as the limited information for those studies identified as “cases taken from medical records” prevents distinguishing if the same scales were used in those studies classified as “self-reported based on validated scale”. In addition, interpretation can differ between physicians and lead to potentially biased results, for example ICD classification allows for subjective interpretation of symptoms [62]. Finally, the effect of *Campylobacter* species was significant for ReA however its true influence is impossible to distinguish as *Campylobacter jejuni* was included in the studies classified as mixed and therefore specific comparisons between the effects of species were not possible. Additionally, truly exploring the effect of species is not possible based on the limited amount of information published on Campylobacter species other than C. *jejuni*. Consequently, despite exploring factors affecting heterogeneity with meta-regression, the majority of this variation remains unexplained as heterogeneity remained high even with sub-group meta-analysis.

The remaining high heterogeneity suggests that potentially influential factors were not captured in this review. This meta-regression focused on study level variables, such as study design, case definitions etc. However host related factors such race, severity of acute illness, the age of *Campylobacter* cases and sequelae cases, medical history or immune status and pathogen related factors such as virulence and dose have been identified as potential influential factors in sequelae development [13, 14, 59]. As a result of the many factors that could potentially affect the likelihood of sequelae development, predicting the proportion of cases of *Campylobacter* that develop chronic sequelae is challenging.

A first step in addressing this challenge would be to increase the accuracy of future estimates through additional high quality data. The effect of lack of reporting of non-results could be influential. In many cases publications reported on the results for a specific sequelae and it was not possible to determine if other sequelae were absent, were not evaluated, or were evaluated but not reported. Additionally, as under-reporting and under-diagnosis of foodborne diseases is well-established [3], there is the potential that cases of sequelae are not being linked to previous infection as these infections have not been identified. For sequelae that can take weeks or months to develop, a previous GI experience may never be linked and therefore not captured in the literature.

The limited information available not only affected our ability to explore heterogeneity in the data, it also raises questions surrounding our current understanding of these diseases. For instance, the link between GBS and campylobacteriosi*s* is widely accepted [4, 5, 15, 16, 18]. The basis for this assumption may be related to the numerous case–control studies published [16] that were excluded from this review because they considered GBS cases with evidence of previous *Campylobacter* exposure. Although this study design indicates a relationship, the lack of temporal relationship and the limited research identified by this systematic review indicate that perhaps the magnitude of the relationship between GBS and *Campylobacter* is not clear. As GBS infection has high mortality, at two to three percent, and 15 to 20 percent of cases develop severe permanent neurological defects [16] further investigation into the role *Campylobacter* infection plays in GBS development is recommended.

In addition, reporting of irritable bowel syndrome as a sequelae linked to *Campylobacter* in burden of disease/cost estimates is inconsistent [9, 10, 63–65]. The results of this systematic review support the inclusion of IBS in BOD estimates for *Campylobacter*. The lack of information available on IBD raises an interesting issue as it is often reported as linked to *Campylobacter* infection within BOD estimates. Our search located only five studies with information related to IBD. Both IBD and IBS are associated with similar symptoms, such as abdominal pain, bloating and diarrhea, with both occurring simultaneously in some cases [60]. This overlap introduces the possibility for misdiagnosis and under diagnosis of these sequelae. Discrepancies between terminology use, case definitions and diagnostic criteria and the lack of information available on the association between IBD and *Campylobacter* infection indicate the need for further research. Further research into the mechanisms of action for sequelae such as GBS, IBS and IBD could help provide more clarity surrounding disease development and links to infection with foodborne pathogens such as *Campylobacter.*

Terminology issues may be a concern with the other sequelae, such as with ReA and Reiter’s syndrome, as terminologies are used interchangeably and there is no standard classification or diagnostic criteria that are universally accepted by the medical community [66]. As many publications did not provide the details of their case definitions for the sequelae and case definitions varied between studies determining the influence this might have on outcome measures was hard to estimate. Without distinct criteria reported within studies, it can be difficult to distinguish which syndromes are actually being assessed and whether they are the same between studies, thereby increasing the complexity involved with identifying relevant studies, contributing to inaccurate estimates and potentially to heterogeneity.

Although the results of this meta-analysis can be used as a starting point to inform burden of disease estimates, the results should be interpreted in the context of the study variables. Inaccessible articles and language restrictions may have prevented the review from having a fully international perspective. Limiting the study results to English language articles prevented the exploration of the effect of country in detail. Additionally, understanding the burden chronic sequelae has in developing countries is an important area for future research. Because multiple outcomes were taken from some studies, the assumption of independence of estimates within studies was not met. For the sequelae that underwent meta-analysis, this could lead to deflated estimates and narrow confidence intervals for the proportion of cases of *Campylobacter* that develop a sequela. Due to the limited amount of data, adjusting for non-independence by averaging the results [66] would have resulted in too few studies to explore with meta-regression. Additionally, the categorization of group size and follow-up time were determined post hoc based on the data. Inconsistencies in the case definitions used for probable cases of *Campylobacter* between studies could also affect the accuracy of the proportions reported. For example, studies using a broader definition for a *Campylobact*er case would result in higher case numbers and a lower proportion for the sequela estimate versus a study with a more stringent definition for a *Campylobacter* case. Many variables varied together consistently across subgroups (for example extra-large populations were often prospective surveillance studies). Combined with these associations, the high heterogeneity and limited number of studies prevented a full meta-regression and sub group meta-analysis therefore all results must be interpreted with caution.

Despite these limitations, the potential burden of long term health complications attributed to these diseases is important considering the high worldwide incidence of campylobacteriosis. Even using the low estimates for the proportion of cases that develop ReA, IBS or GBS, there is the potential for tens of thousands of sequelae cases yearly in North America alone. Considering the long term nature and potential severity of these associated health complications, this could contribute to a significant decrease in quality of life and a significant burden on health care systems worldwide.

## Conclusions

The proportion of *Campylobacter* cases developing chronic sequelae varied greatly depending on a variety of factors, not all of which were identified in this review. Estimates for the proportion of cases of reactive arthritis, irritable bowel syndrome and Guillain Barré syndrome were, 2.86% (95% CI 1.4% - 5.61%), 4.01% (95% CI 1.41% - 10.88%) and 0.07% (95% CI. 0.03% - 0.15) respectively. These results should be interpreted with caution due to the high heterogeneity and limited data which prevented detailed exploration of sources of heterogeneity. Although these are the best estimates currently available based on all international sources, in order for more accurate estimates to be developed exploration of non-English language studies is recommended and consistent diagnostic approaches and case definitions need to be implemented and reported in future research.

## Electronic supplementary material

Additional file 1: **PRISMA 2009 Flow Diagram Campylobacter SLR.** (DOC 64 KB)

## Abbreviations

AGI: Acute gastrointestinal illness
REA: Reactive arthritis
RS: Reiter’s syndrome
HUS: Haemolytic uraemic syndrome
IBS: Irritable bowel syndrome
IBD: Inflammatory bowel disease
GBS: Guillain Barré syndrome
MFS: Miller Fisher syndrome
ONBOIDS: Ontario Burden of Infectious Disease Study
UC: Ulcerative colitis
Crohn’s: Crohn’s disease.

## References

[1] Scallan E, Hoekstra RM, Angulo FG, Tauxe RV, Widdowson MA, Roy SL, Jones JL, Griffin PM (2011). Foodborne illness acquired in the United States - major pathogens. Emerg Infect Dis.

[2] Government of Canada (2012). Canadian Notifiable Disease Surveillance System National Report (2005–2008).

[3] Thomas MK, Majowicz SE, Sockett PN, Fazil A, Pollari F, Doré K, Flint JA, Edge VL (2006). Estimated numbers of community cases of illness due to *Salmonella*, *Campylobacter* and verotoxigenic *Escherichia coli*: Pathogen-specific community rates. Can J Infect Dis Med Microbiol.

[4] Smith JL (2002). *Campylobacter jejuni* infection during pregnancy: Long-term consequences of associated bacteremia, Guillain-Barré syndrome, and reactive arthritis. J Food Prot.

[5] Peterson MC (1994). Clinical aspects of *Campylobacter jejuni* infections in adults. West J Med.

[6] Abley MJ, Wittum TE, Funk JA, Gebreyes WA (2012). Antimicrobial susceptibility, pulsed-field gel electrophoresis, and multi-locus sequence typing of *Campylobacter coli* in swine before, during, and after the slaughter process. Foodborne Pathog Dis.

[7] Hermans D, Pasmans F, Messens W, Martel A, Van Immerseel F, Rasschaert G, Heyndrickx M, Van Deun K, Haesebrouck F (2012). Poultry as a host for the zoonotic pathogen *Campylobacter jejuni*. Vector Borne Zoonotic Dis.

[8] Domingues AR, Pires SM, Halasa T, Hald T (2012). Source attribution of human campylobacteriosis using a meta-analysis of case–control studies of sporadic infections. Epidemiol Infect.

[9] Kwong JC, Crowcroft NS, Campitelli MA, Ratnasingham S, Daneman N, Deeks SL, Manuel DG (2010). Ontario burden of infectious disease study (ONBOIDS): An OAHPP/ICES report.

[10] Havelaar AH, Haagsma JA, Mangen MJ, Kemmeren JM, Verhoef LP, Vijgen SM, Wilson M, Friesema IH, Kortbeek LM, van Duynhoven YT, van Pelt W (2012). Disease burden of foodborne pathogens in the Netherlands, 2009. Int J Food Microbiol.

[11] Liberati A, Altman DG, Tetzlaff J, Mulrow C, Gøtzsche PC, Ioannidis JP, Clarke M, Devereaux PJ, Kleijnen J, Moher D (2009). The PRISMA statement for reporting systematic reviews and meta-analysis of studies that evaluate health care interventions. Br Med J.

[12] Higgins JPT, Green S (2011). Cochrane handbook for systematic reviews of interventions version 5.1.0. Cochrane Collaboration.

[13] Thabane M, Kottachchi DT, Marshall JK (2007). Systematic review and meta-analysis: The incidence and prognosis of post-infectious irritable bowel syndrome. Aliment Pharmacol Ther.

[14] Karlinger K, Gyorke T, Mako E, Mester A, Tarján Z (2000). The epidemiology and the pathogenesis of inflammatory bowel disease. Eur J Radiol.

[15] Israeli E, Agmon-Levin N, Blank M, Chapman J, Shoenfeld Y (2012). Guillain-Barré syndrome–a classical autoimmune disease triggered by infection or vaccination. Clin Rev Allergy Immunol.

[16] Nachamkin I, Allos BM, Ho T (1998). *Campylobacter* species and Guillain-Barré syndrome. Clin Microbiol Rev.

[17] Pope JE, Krizova A, Garg AX, Thiessen-Philbrook H, Ouimet JM (2007). *Campylobacter* reactive arthritis: A systematic review. Semin Arthritis Rheum.

[18] Poropatich KO, Walker CL, Black RE (2010). Quantifying the association between *Campylobacter* infection and Guillain-Barré syndrome: A systematic review. J Health Popul Nutr.

[19] Riddle MS, Gutierrez RL, Verdu EF, Porter CK (2012). The chronic gastrointestinal consequences associated with *Campylobacter*. Curr Gastroenterol Rep.

[20] Sanchez J, Dohoo IR, Christensen J, Rajic A (2007). Factors influencing the prevalence of *Salmonella* spp. in swine farms: A meta-analysis approach. Prev Vet Med.

[21] Egger M, Smith D, Altmand DG (2001). Systematic reviews in health care: Meta-analysis in context.

[22] Higgins JP, Thompson SG (2002). Quantifying heterogeneity in a meta-analysis. Stat Med.

[23] Pitkanen T, Pettersson T, Ponka A, Kosunen TU (1981). Clinical and serological studies in patients with *Campylobacter fetus/ssp*/*jejuni* infection: clinical findings. Infection.

[24] Wang SC, Chang LY, Hsueh PR, Lu CY, Lee PI, Shao PL, Hsieh YC, Yen FP, Lee CY, Huang LM (2008). Campylobacter enteritis in children in northern Taiwan–a 7-year experience. J Microbiol Immunol Infect.

[25] Short CD, Klouda PT, Smith L (1982). *Campylobacter jejuni*/*enteritis* and reactive arthritis. Ann Rheum Dis.

[26] Rowe PC, Walop W, Lior H, Mackenzie AM (1991). Haemolytic anaemia after childhood Escherichia coli O 157.H7 infection: Are females at increased risk?. Epidemiol Infect.

[27] Petersen AM, Nielsen SV, Meyer D, Ganer P, Ladefoged K (1996). Bacterial gastroenteritis among hospitalized patients in a Danish county, 1991–93. Scand J Gastroenterol.

[28] Saps M, Pensabene L, Di Martino L, Staiano A, Wechsler J, Zheng X, Di Lorenzo C (2008). Post-infectious functional gastrointestinal disorders in children. J Pediatr.

[29] Pitkänen T, Pönkä A, Peterson T, Kosunen TU (1983). *Campylobacter enteritis* in 188 hospitalized patients. Arch Intern Med.

[30] Spiller RC, Jenkins D, Thornley JP, Hebden JM, Wright T, Skinner M, Neal KR (2000). Increased rectal mucosal enteroendocrine cells, T lymphocytes, and increased gut permeability following acute *Campylobacter enteritis* and in post-dysenteric irritable bowel syndrome. Gut.

[31] Spence MJ, Moss-Morris R (2007). The cognitive behavioral model of irritable bowel syndrome: A prospective investigation of patients with gastroenteritis. Gut.

[32] Melby K, Dahl OP, Crisp L, Penner JL (1990). Clinical and serological manifestations in patients during a waterborne epidemic due to *Campylobacter jejuni*. J Infect.

[33] Bremell T, Bjelle A, Svedhem A (1991). Rheumatic symptoms following an outbreak of *Campylobacter enteritis*: A five year follow up. Ann Rheum Dis.

[34] Gardner TJ, Fitzgerald C, Xavier C, Klein R, Pruckler J, Stroika S, McLaughlin JB (2011). Outbreak of campylobacteriosis associated with consumption of raw peas. Clin Infect Dis.

[35] Eastmond CJ, Rennie JA, Reid TM (1983). An outbreak of Campylobacter enteritis–a rheumatological follow up survey. J Rheumatol.

[36] McCarthy N, Andersson Y, Jormanainen V, Gustavsson O, Giesecke J (1999). The risk of Guillain-Barré syndrome following infection with *Campylobacter jejuni*. Epidemiol Infect.

[37] Locht H, Krogfelt KA (2002). Comparison of rheumatological and gastrointestinal symptoms after infection with *Campylobacter jejuni*/*coli* and enterotoxigenic *Escherichia coli*. Ann Rheum Dis.

[38] Helms M, Simonsen J, Molbak K (2006). Foodborne bacterial infection and hospitalization: A registry-based study. Clin Infect Dis.

[39] Kosunen TU, Ponka A, Kauranen O, Martio J, Pitkänen T, Hortling L, Aittoniemi S, Penttilä O, Koskimies S (1981). Arthritis associated with *Campylobacter jejuni*/*enteritis*. Scand J Rheumatol.

[40] Ponka A, Pitkanen T, Sarna S, Kosunen TU (1984). Infection due to *Campylobacter jejuni*: A report of 524 outpatients. Infection.

[41] Schiellerup P, Krogfelt KA, Locht H (2008). A comparison of self-reported joint symptoms following infection with different enteric pathogens: Effect of HLA-B27. J Rheumatol.

[42] Dunlop SP, Jenkins D, Neal KR, Spiller RC (2003). Relative importance of enterochromaffin cell hyperplasia, anxiety, and depression in post infectious IBS. Gastroenterology.

[43] Hannu T, Mattila L, Rautelin H, Pelkonen P, Lahdenne P, Siitonen A, Leirisalo-Repo M (2002). *Campylobacter*-triggered reactive arthritis: A population-based study. Rheumatology.

[44] Doorduyn Y, Van Pelt W, Siezen CL, Van Der Horst F, Van Duynhoven YT, Hoebee B, Janssen R (2008). Novel insight in the association between salmonellosis or campylobacteriosis and chronic illness, and the role of host genetics in susceptibility to these diseases. Epidemiol Infect.

[45] Moss-Morris R, Spence M (2006). To “lump” or to “split” the functional somatic syndromes: Can infectious and emotional risk factors differentiate between the onset of chronic fatigue syndrome and irritable bowel syndrome?. Psychosom Med.

[46] Thornley JP, Jenkins D, Neal K, Wright T, Brough J, Spiller RC (2001). Relationship of *Campylobacter* toxigenicity in vitro to the development of post infectious irritable bowel syndrome. J Infect Dis.

[47] Townes JM, Deodhar AA, Laine ES, Smith K, Krug HE, Barkhuizen A, Thompson ME, Cieslak PR, Sobel J (2008). Reactive arthritis following culture-confirmed infections with bacterial enteric pathogens in Minnesota and Oregon: A population-based study. Ann Rheum Dis.

[48] Jess T, Simonsen J, Nielsen NM, Jørgensen KT, Bager P, Ethelberg S, Frisch M (2011). Enteric *Salmonella* or *Campylobacter* infections and the risk of inflammatory bowel disease. Gut.

[49] Gumpel JM, Martin C, Sanderson PJ (1981). Reactive arthritis associated with *Campylobacter enteritis*. Ann Rheum Dis.

[50] Tam CC, Rodrigues LC, Petersen I, Islam A, Hayward A, O'Brien SJ (2006). Incidence of Guillain-Barré syndrome among patients with *Campylobacter* infection: A general practice research database study. J Infect Dis.

[51] Schoenberg-Norio D, Mattila L, Lauhio A, Katila ML, Kaukoranta SS, Koskela M, Pajarre S, Uksila J, Eerola E, Sarna S, Rautelin H (2010). Patient-reported complications associated with *Campylobacter jejuni* infection. Epidemiol Infect.

[52] Ternhag A, Torner A, Svensson A, Ekdahl K, Giesecke J (2008). Short- and long-term effects of bacterial gastrointestinal infections. Emerg Infect Dis.

[53] McCarthy N, Giesecke J (2001). Incidence of Guillain-Barré syndrome following infection with *Campylobacter jejuni*. Am J Epidemiol.

[54] Hannu T, Kauppi M, Tuomala M, Laaksonen I, Klemets P, Kuusi M (2004). Reactive arthritis following an outbreak of *Campylobacter jejuni* infection. J Rheumatol.

[55] Melby KK, Svendby JG, Eggebo T, Holmen LA, Andersen BM, Lind L, Sjøgren E, Kaijser B (2000). Outbreak of ***Campylobacter*****infection in a subarctic community**. Eur J Clin Microbiol Infect Dis.

[56] Thompson WG, Creed F, Drossman DA, Heaton KW, Mazzacca G (1992). Functional bowel disease and functional abdominal pain. Gastroenterol Int.

[57] Thompson WG, Longstreth GF, Drossman DA, Heaton KW, Irvine EJ, Muller-Lissner SA (1999). Functional bowel disorders and functional abdominal pain. Gut.

[58] Svedlund J, Sjodin I, Dotevall G (1988). GSRS–a clinical rating scale for gastrointestinal symptoms in patients with irritable bowel syndrome and peptic ulcer disease. Dig Dis Sci.

[59] Hughes RA, Hadden RD, Gregson NA, Smith KJ (1999). Pathogenesis of Guillain-Barré syndrome. J Neuroimmunol.

[60] Ohman L, Simren M (2010). Pathogenesis of IBS: Role of inflammation, immunity and neuroimmune interactions. Gastroenterol Hepatol.

[61] Townes JM (2010). Reactive arthritis after enteric infections in the United States: The problem of definition. Clin Infect Dis.

[62] World Health Organization (2012). International classification of Diseases.

[63] Withington SG, Chambers ST (1997). The cost of campylobacteriosis in New Zealand in 1995. N Z Med J.

[64] Mangen MJ, Havelaar AH, Bernsen R, Van Koningsveld R, De Wit GA (2005). The costs of human *Campylobacter* infections and sequelae in the Netherlands: A DALY and cost-of-illness approach. Food Econ.

[65] Ruzante JM, Majowicz SE, Fazil A, Davidson VJ (2011). Hospitalization and deaths for select enteric illnesses and associated sequelae in Canada, 2001–2004. Epidemiol Infect.

[66] Borenstein M, Hedges LV, Higgins JPT, Rothstein HR (2009). Introduction to meta-analysis.

[CR67] The pre-publication history for this paper can be accessed here: http://www.biomedcentral.com/1471-2458/14/1203/prepub

